# Composite Haematological and Nutritional Indices Show No Evidence of Improved Long-Term Mortality Discrimination Beyond Their Own Components in Non-ST-Segment Elevation Myocardial Infarction

**DOI:** 10.3390/medicina62101965

**Published:** 2026-10-11

**Authors:** Emrah Bozbeyoğlu, Aylin Sungur, Fatma Can, Barış Şimşek, Duygu Genç Albayrak, Funda Özlem Pamuk, Can Yücel Karabay

**Affiliations:** 1 Department of Cardiology, Dr. Siyami Ersek Thoracic and Cardiovascular Surgery Training and Research Hospital, University of Health Sciences, Istanbul 34668, Türkiye; 2 Department of Cardiology, Basaksehir Cam and Sakura City Hospital, Istanbul 34480, Türkiye; 3 Department of Cardiology, Sureyyapasa Chest Diseases and Thoracic Surgery Training and Research Hospital, University of Health Sciences, Istanbul 34844, Türkiye; 4 Department of Cardiology, Faculty of Medicine, Yeditepe University, Istanbul 34755, Türkiye

**Keywords:** non-ST-segment elevation myocardial infarction, prognosis, inflammation, composite biomarkers, C-reactive protein, albumin, discrimination

## Abstract

*Background and Objectives*: Composite indices from routine inflammatory, haematological and nutritional measurements are widely reported as prognostic markers after acute coronary syndromes, but whether they add information beyond their components is uncertain. *Materials and Methods*: In 30-day survivors of non-ST-segment elevation myocardial infarction (NSTEMI) undergoing coronary angiography at a single centre, thirteen composite indices and six prespecified components were evaluated for five-year all-cause mortality using ROC analysis, Cox regression and the change in Harrell’s C-index (ΔC) over a study clinical model. Each index was also compared with a freely weighted model of its own constituents and with a reference model adding CRP and albumin. *Results*: Among 561 patients (median follow-up 5.48 years), 94 died, 86 within five years. Composite-index AUCs ranged from 0.616 to 0.689; none exceeded CRP alone after multiplicity correction. Eight indices were nominally associated with mortality after adjustment (hazard ratios 1.26–1.38 per 1 SD), but every bootstrap confidence interval for ΔC over the clinical model included zero. Fixed indices and freely weighted constituents performed similarly (cross-validated ΔC −0.0089 to +0.0053), with one small exploratory difference favouring the fixed MLR ratio. Adding CRP and albumin raised the C-index from 0.7720 to 0.7826; over this reference, no estimable composite showed evidence of association (all *p* ≥ 0.19). *Conclusions*: In 30-day survivors of NSTEMI, composite indices showed no evidence of improved long-term mortality discrimination over a study-specific clinical model and contained no information beyond their measured constituents. Their independent associations may largely reflect information already present in the components. Small gains cannot be excluded; the findings do not establish equivalence.

## 1. Introduction

Long-term mortality after non-ST-segment elevation myocardial infarction (NSTEMI) remains substantial despite contemporary management [1,2,3], and identifying in advance the patients in whom it will occur remains difficult. This difficulty has motivated a search for inexpensive prognostic markers that can be obtained from admission blood work.

A large and rapidly growing literature now reports that composite indices calculated from routine haematological and biochemical measurements predict outcome after acute coronary syndromes. The haemoglobin–albumin–lymphocyte–platelet (HALP) score, originally developed in gastric cancer [4], has been associated with in-hospital and one-year mortality in NSTEMI and with no-reflow and short-term mortality in ST-segment elevation myocardial infarction [5,6,7]. A modified HALP score has been proposed on the premise that a low rather than a high platelet count is the more informative marker [8]. The C-reactive protein–albumin–lymphocyte (CALLY) index has been reported as a predictor of mortality in acute coronary syndromes and after primary percutaneous coronary intervention [9,10,11], and the C-reactive protein-to-albumin ratio (CAR) as a predictor of in-hospital mortality in the same setting [12]. The systemic immune-inflammation index (SII) has been associated with long-term cardiovascular mortality after NSTEMI [13] and the systemic inflammation response index (SIRI) with major adverse cardiac events in the same population [14], while simple haemogram-derived ratios, among them the neutrophil-to-lymphocyte ratio (NLR) and the CRP-to-lymphocyte ratio (CLR), have likewise been linked to adverse events after NSTEMI [15]. The neutrophil percentage-to-albumin ratio (NPAR) has been described in the same setting [16], and the prognostic performance of several of these markers has recently been shown to depend on the time horizon considered [17].

These indices share an important structural property that has received limited attention: they are algebraic recombinations of a small pool of shared measurements—essentially haemoglobin, albumin, C-reactive protein (CRP) and the differential leucocyte and platelet counts. HALP and PNI share albumin and lymphocytes; CALLY, CAR and CLR share CRP; SII, NLR and the pan-immune-inflammation value share the neutrophil and lymphocyte counts. A composite can therefore appear to be an independent predictor simply because one of its constituents is prognostically important, and two indices can appear to be different markers while carrying nearly identical information. Most published NSTEMI analyses have evaluated composites against clinical covariates or individual markers rather than against models containing all of their own constituents jointly. Partial constituent comparisons exist—for example, NPAR has been compared directly with albumin and neutrophils [16]—and a recent study in rheumatoid arthritis compared CALLY with CRP, albumin and lymphocyte count entered together [18]. To our knowledge, however, this question has not been examined systematically across a broad panel of composite indices in NSTEMI.

We therefore examined a panel of thirteen composite indices and six prespecified individual components in a single NSTEMI cohort with completely observed five-year vital status; monocyte count and total white blood cell count were additionally used only where required to reconstruct specific indices. Our aim was not to identify the best index but to ask a prior question: does combining these routine measurements into a composite provide prognostic information beyond a clinical model, and beyond the measurements themselves?

## 2. Materials and Methods

### 2.1. Study Population

We retrospectively analysed an established institutional registry of consecutive patients with NSTEMI who underwent coronary angiography at Dr Siyami Ersek Training and Research Hospital, University of Health Sciences, Istanbul, from May 2017 through May 2018. NSTEMI was defined according to the European Society of Cardiology guidance applicable during the enrolment period.

The registry contained 748 records. We excluded 26 records without a valid admission date. Duplicate entries were identified for eight patients and resolved before outcome analysis by retaining the earliest documented admission as the index event; when records shared the same admission date, the more complete entry was retained. This procedure removed seven records. To examine long-term rather than acute-phase prognosis, the analysis was landmarked at 30 days, resulting in the exclusion of five early deaths. We additionally excluded five patients aged >85 years and 19 with active malignancy. Among the remaining 686 otherwise eligible patients, 125 did not meet the laboratory-completeness criterion required for the prespecified complete-panel comparison (albumin unavailable in 60, CRP in 39, and complete blood count with differential in 34; categories overlap). The final analytic cohort therefore comprised 561 patients with the laboratory measurements required to calculate the complete index panel (Supplementary Figure S1).

The data come from an institutional acute coronary syndrome registry at Istanbul Dr Siyami Ersek Thoracic and Cardiovascular Surgery Training and Research Hospital, where research applications were at that time reviewed by the hospital’s Medical Specialty and Education Board. The board granted research permission for the analysis of acute coronary syndrome patients at this institution on 25 May 2018 (document no. 28001928-508.01). The present study is a secondary analysis of the same anonymised registry, and five-year vital status was obtained by linkage to the national death notification system. Individual informed consent was not obtained, the study being a retrospective analysis of existing anonymised records. The signed permission document is available to the editorial office on request, and we are happy to supply any further documentation the journal requires.

The underlying institutional NSTEMI registry has previously been used for ECG-based investigations of acute coronary occlusion and specific electrocardiographic patterns [19,20]. It also overlaps with a separate analysis comparing the CHA_2_DS_2_-VA and CHA_2_DS_2_-VASc scores that is currently under consideration elsewhere. That analysis addresses different exposures and a different research question and uses a different analytic population; the overlap is disclosed here and in the accompanying cover letter.

### 2.2. Laboratory Measurements and Index Definitions

Venous blood samples obtained within 24 h of admission were analysed in the hospital laboratory. Complete blood counts were measured on a Mindray BC-6800 automated haematology analyser (Mindray, Shenzhen, China); serum albumin was measured by a colorimetric bromocresol green assay and CRP by immunoturbidimetry on a Roche Cobas 6000 analyser (Roche Diagnostics, Mannheim, Germany).

Thirteen composite indices were computed (definitions in Section 3.2): HALP, modified HALP, CALLY, CAR, CLR, PNI, NPAR, SII, the systemic inflammation response index (SIRI), the pan-immune-inflammation value (PIV), NLR, the platelet-to-lymphocyte ratio (PLR) and the monocyte-to-lymphocyte ratio (MLR). Six prespecified individual components were analysed in parallel as standalone markers: CRP, albumin, lymphocyte count, haemoglobin, platelet count and neutrophil count. Monocyte count and total white blood cell count were used additionally only where required to reconstruct SIRI, PIV, MLR and NPAR for the own-constituent analyses; they were not part of the prespecified standalone ROC comparison panel. All indices and standalone components were logarithmically transformed and standardised, and signs were oriented so that a one standard deviation increase always denotes an adverse change; hazard ratios are therefore directly comparable across markers. PNI is the one exception to this rule in the constituent analysis: it is an additive combination of albumin and the lymphocyte count on the raw scale rather than a multiplicative one, so for that comparison, PNI and its two components were standardised on the raw scale. All other analyses use the log-transformed value throughout, which is why the C-index reported for PNI differs slightly between the incremental-discrimination and own-constituent analyses.

### 2.3. Outcome and Follow-Up

All-cause mortality after the 30-day landmark was the primary outcome, with survival time calculated from the index admission. Administrative follow-up ended on 1 June 2023. Every surviving patient had at least 5.14 years of observation; consequently, vital status at five years was known for the entire analytic cohort and the fixed-horizon discrimination analyses did not require adjustment for censoring. Time-to-event models incorporated the full duration of available follow-up.

### 2.4. Statistical Analysis

Categorical variables are presented as counts with percentages and compared with the chi-square test or, when an expected cell count was below five, Fisher’s exact test. Continuous variables are presented as mean ± standard deviation or median with interquartile range according to distribution and compared with the independent-samples t-test or the Mann–Whitney U test.

Discrimination was quantified by the area under the receiver operating characteristic curve (AUC) for mortality at one, three and five years, all of which were completely observed; the five-year endpoint was the primary horizon. Confidence intervals were derived by the method of DeLong, and each marker was compared with CRP alone using the DeLong test for correlated curves [21]. CRP was designated as the comparator because it is the single most widely available inflammatory measurement and is a constituent of three of the composites examined (CALLY, CAR and CLR).

Cox proportional-hazards models were fitted unadjusted and adjusted for a prespecified clinical covariate model fixed before the marker comparisons were run, comprising age, male sex, diabetes mellitus, hypertension, prior myocardial infarction, left ventricular ejection fraction, estimated glomerular filtration rate, Killip class ≥ 2, multivessel disease and revascularisation. The study clinical model was used as an adjustment and discrimination benchmark rather than as a deployable admission-time prediction score. Revascularisation was included because it is strongly associated with survival in this cohort, but it is a post-admission treatment variable that could in principle lie on the causal path between an inflammatory marker and death; a prespecified sensitivity analysis omitted it. Because multivessel disease and revascularisation are determined after initial presentation, an additional admission-oriented sensitivity model omitted both variables. The proportional-hazards assumption was assessed with scaled Schoenfeld residuals for the common clinical-plus-CRP-and-albumin reference model. Possible non-linearity was examined for CRP and albumin, the two laboratory terms used in that common reference model, using restricted cubic splines with three degrees of freedom and likelihood-ratio tests against the corresponding linear specification. These checks were not intended as exhaustive assumption testing for every marker-specific Cox model.

Incremental discrimination was quantified as the change in Harrell C-index when a marker was added to the clinical model, with percentile confidence intervals and optimism correction by the bootstrap method of Harrell [22], both based on 2000 resamples drawn in common across markers so that the estimates are directly comparable. Because the C-index is known to be insensitive to added predictors, the likelihood-ratio test for the same addition is reported alongside it, so that model fit and discrimination can be read against one another. Calibration was examined by comparing predicted and observed five-year mortality across fifths of predicted risk. Each index was then compared with a model containing the clinical covariates plus its own constituents entered separately with freely estimated coefficients. Because every index in the panel is a fixed-weight combination of its constituents, the model containing the index is a restricted version of the model containing its components, and the two were compared by likelihood-ratio test on k − 1 degrees of freedom, where k is the number of constituents. This own-constituent comparison was performed in the maximal marker-specific sample containing the relevant index, its constituents, outcome and clinical covariates, so that an unrelated missing laboratory measurement did not exclude a patient. Relative predictive performance was internally validated with five repeats of stratified five-fold cross-validation. Each patient’s out-of-fold linear predictor was averaged across repeats; the paired difference in cross-validated C-index was defined as the constituent-model C-index minus the fixed-index C-index, and a 2000-resample patient-level bootstrap of the paired cross-validated prediction vectors provided its 95% confidence interval. This bootstrap resampled the generated cross-validated prediction vectors rather than repeating the complete cross-validation and model-fitting procedure within each resample; the resulting intervals therefore quantify uncertainty conditional on those cross-validated predictions and are interpreted descriptively. Finally, as an empirical decomposition analysis, the models were repeated against a common reference consisting of the clinical covariates plus CRP and albumin entered as separate terms. CRP and albumin were chosen because they are shared constituents of several leading indices and provide a simple common benchmark; this reference model was not an externally validated risk score and its selection should be interpreted as empirical rather than independently prespecified.

Analyses were complete-case with no imputation. For the primary common-sample multivariable analyses, the only covariate with missing data in the clinical model was estimated glomerular filtration rate (43 patients, 7.7%), so these analyses use 518 patients with 84 deaths. The marker-specific own-constituent comparison instead used 518 to 606 patients with 84 to 92 deaths, depending on the laboratory measurements required by each index. A raw missingness audit confirmed that the binary covariates used in the clinical model had no missing values before indicator coding. Four prespecified sensitivity analyses were performed: the fixed five-year endpoint instead of complete follow-up; retention of duplicate records; exclusion of patients with non-obstructive coronary arteries; and omission of revascularisation from the clinical model. An additional admission-oriented sensitivity analysis omitted both multivessel disease and revascularisation. A post hoc sensitivity analysis replaced any revascularisation with a three-level revascularisation strategy: no revascularisation; percutaneous coronary intervention of the culprit vessel only in multivessel disease; and other revascularisation (revascularisation in single-vessel disease, coronary artery bypass grafting, or percutaneous coronary intervention of more than one vessel at the index procedure or as a staged procedure). For this model, ΔC confidence intervals were obtained from 2000 bootstrap resamples; resamples in which any Cox model failed to converge (16 of 2016 drawn) were replaced. Each sensitivity specification was applied to all twelve indices that are estimable in the reference model and is reported in Supplementary Table S4. In addition, a marker-specific available-case sensitivity analysis re-fitted each composite in all otherwise eligible patients with that marker and all clinical covariates observed (Supplementary Table S2), and a marker/reference-specific available-case analysis repeated the CRP-plus-albumin decomposition in all patients with the relevant composite, CRP, albumin, outcome and clinical covariates observed (Supplementary Table S3). For the primary CRP-plus-albumin decomposition, uncertainty in ΔC was quantified by a 1000-resample full-refit paired patient-level bootstrap using common resamples across the twelve estimable composites. The study was not registered and no protocol was deposited; the analysis plan was fixed before the marker comparisons were run, but this cannot be independently verified. Marker-wise Cox and own-constituent likelihood-ratio *p* values are reported as nominal and were not adjusted for multiplicity; the DeLong comparison family is interpreted against the prespecified Bonferroni threshold reported below. Primary analyses were performed with Python 3.12.3 (lifelines 0.30.3, SciPy 1.17.1 and scikit-learn 1.8.0); the extended bootstrap, cross-validation and robustness checks were independently reproduced with statsmodels PHReg 0.14.6; the post hoc revascularisation-strategy analysis was performed with statsmodels PHReg 0.15.0 (Efron ties).

## 3. Results

### 3.1. Cohort

The 561 patients had a mean age of 59.4 ± 10.7 years and 433 (77.2%) were men. Hypertension was present in 310 (55.3%) and diabetes in 174 (31.0%). Coronary anatomy showed non-obstructive disease in 92 (16.4%) and multivessel disease in 220 (39.2%); 375 patients (66.8%) were revascularised. Over a median follow-up of 5.48 years (interquartile range 5.25–5.65), 94 patients (16.8%) died, 86 of them (15.3%) within five years.

Baseline characteristics according to five-year survival are shown in Table 1. Non-survivors had a heavier clinical risk burden: they were older, more often had hypertension, diabetes, prior myocardial infarction and prior stroke, had a higher heart rate, lower ejection fraction and lower estimated glomerular filtration rate, and were less often revascularised. Among patients with multivessel disease, multivessel revascularisation (coronary artery bypass grafting, or percutaneous coronary intervention of more than one vessel) was performed in 8 of 38 non-survivors (21.1%) and 99 of 182 survivors (54.4%; *p* < 0.001). All thirteen composite indices and five of the six components differed significantly between survivors and non-survivors. The single exception was the platelet count, which was virtually identical in the two groups (239.9 ± 64.4 versus 241.8 ± 82.5 × 10^9^/L, *p* = 0.838).

### 3.2. Discrimination for Five-Year Mortality

**Figure 1:**
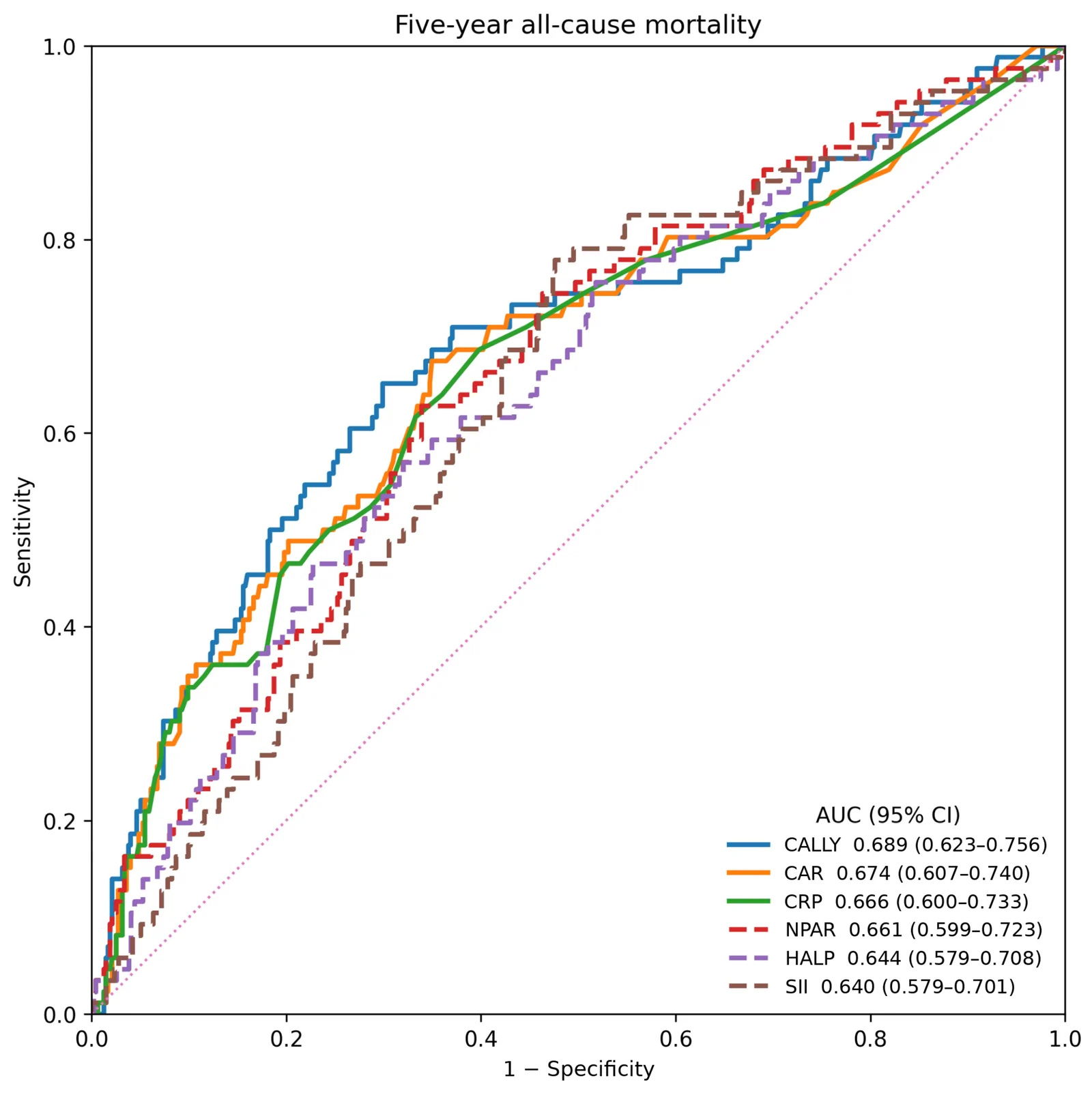
Receiver operating characteristic curves for five-year all-cause mortality. Curves are shown for the two indices with nominally higher areas under the curve than C-reactive protein before multiplicity correction (CALLY, CAR), for C-reactive protein itself, and for three widely reported indices (NPAR, HALP, SII). Areas under the curve with 95% confidence intervals are given in the legend. The dotted diagonal line indicates no discrimination (area under the curve 0.5).

Areas under the curve are shown in Table 2 and Figure 1. Discrimination was modest and closely clustered across the panel, ranging from 0.616 for PIV to 0.689 for CALLY. CRP alone achieved an AUC of 0.666 (95% CI 0.600–0.733). Two indices had nominally higher AUCs than CRP alone: CALLY (difference +0.023, DeLong *p* = 0.035) and CAR (+0.007, *p* = 0.043). Both differences are small in absolute terms, and neither *p* value survives Bonferroni correction for the eighteen pairwise comparisons made against CRP (threshold 0.0028), so these results are best read as showing that the panel is closely clustered rather than that either index is superior. Of the remaining sixteen markers, none differed from CRP, including CLR (0.685; difference +0.018, *p* = 0.080), with the single exception of the platelet count, which was significantly worse (−0.180, *p* < 0.001). Platelet count alone had an AUC of 0.486 (95% CI 0.418–0.554), providing no evidence of useful univariable discrimination, which is directly relevant to the interpretation of the two HALP variants, which incorporate it as a divisor (HALP) or as a multiplier (modified HALP).

### 3.3. Association with Mortality After Adjustment

In unadjusted Cox models, every marker except the platelet count was associated with mortality (Table 3). After adjustment for the clinical model, eight of the thirteen composite indices were nominally associated with all-cause mortality, with adjusted hazard ratios between 1.26 and 1.38 per one standard deviation adverse change: CAR 1.38 (95% CI 1.12–1.69), NPAR 1.36 (1.07–1.73), CALLY 1.34 (1.09–1.65), CLR 1.32 (1.07–1.62), SII 1.29 (1.03–1.61), HALP 1.29 (1.02–1.64), PNI 1.29 (1.01–1.65) and PIV 1.26 (1.01–1.57). These marker-wise *p* values are nominal and are not multiplicity-adjusted. NLR, SIRI, PLR, MLR and the modified HALP score were not associated after adjustment. Among the individual components, CRP (1.35, 1.10–1.66), albumin (1.27, 1.07–1.51), neutrophil count (1.32, 1.06–1.64) and haemoglobin (1.28, 1.03–1.60) had nominal adjusted associations, whereas the lymphocyte count (1.04, 0.81–1.33, *p* = 0.759) and the platelet count (1.20, 0.97–1.48, *p* = 0.093) did not.

For the common clinical-plus-CRP-and-albumin reference model, the proportional-hazards test showed no evidence of violation (minimum covariate *p* = 0.116; global test χ^2^ = 8.88, *p* = 0.713). Restricted cubic splines showed no evidence of departure from linearity on the logarithmic scale for CRP (non-linearity *p* = 0.552) or albumin (*p* = 0.820); the overall adjusted association was significant for CRP (*p* = 0.029) but not for albumin (*p* = 0.096). These results apply to the tested reference-model terms and should not be read as comprehensive assumption testing for every marker-specific model.

This is the point at which most published analyses of these indices conclude. Read in isolation, Table 3 supports the conventional message that several composite indices are independent predictors of long-term mortality in NSTEMI.

### 3.4. Persistence of Discrimination over Time

Mortality at one, three and five years (23, 48 and 86 deaths respectively) was completely observed, so discrimination could be examined at each horizon without censoring adjustment (Supplementary Table S5). The CRP-containing markers discriminated somewhat better at one year than at five, whereas NLR, NPAR and SII performed marginally better at the later horizon; the ordering of markers was broadly but not entirely preserved, albumin falling by four ranks and NPAR rising by three between the two horizons among the markers shown. With only 23 deaths at one year the confidence intervals at that horizon span roughly 0.3 of area, so these comparisons are descriptive and no formal test of a time trend in discrimination was performed. CALLY and CLR tracked one another almost exactly at every horizon (one year 0.723 versus 0.721; three years 0.665 versus 0.662; five years 0.689 versus 0.685), an observation that is explained in the following section.

### 3.5. Incremental Discrimination over the Clinical Model

**Figure 2:**
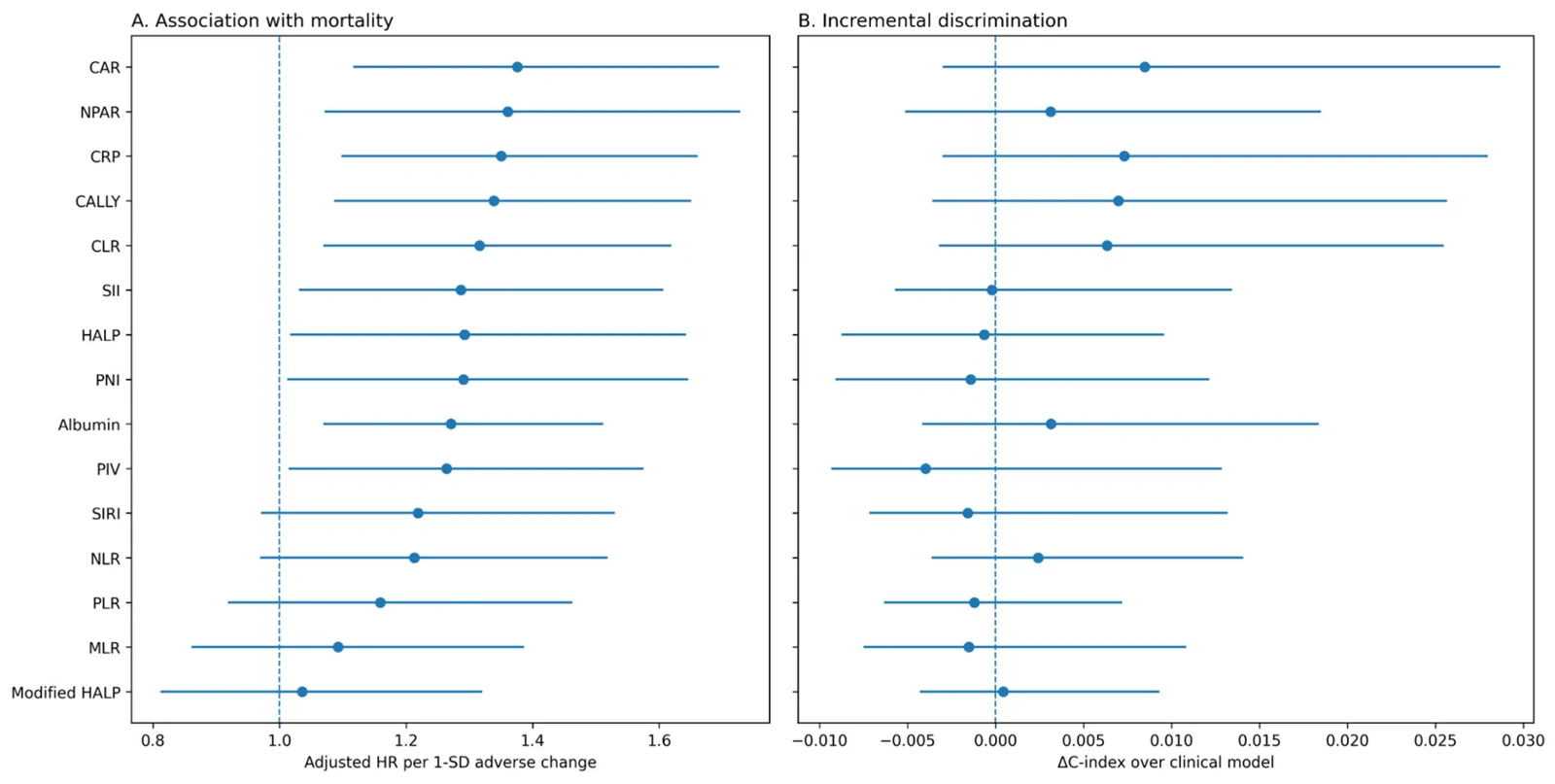
Association with mortality versus incremental discrimination. (**A**) Adjusted hazard ratios per one standard deviation adverse change for all thirteen composite indices plus CRP and albumin, from Cox models containing the clinical model, with 95% confidence intervals. (**B**) Change in Harrell’s C-index when the same marker is added to the clinical model, with 2000-resample bootstrap 95% confidence intervals; no interval excludes zero. Vertical dashed lines mark the null values (hazard ratio of 1 in panel A; ΔC of 0 in panel B). The contrast between the two panels is the principal finding of the study.

The clinical model alone achieved a C-index of 0.7720 in the primary implementation (0.7721 in the independent statsmodels re-analysis). Adding each of the thirteen composite indices, CRP or albumin produced apparent changes in the C-index between −0.0040 and +0.0085 (Table 4, Figure 2B). The largest increments were observed for CAR (+0.0085), CRP (+0.0073) and CALLY (+0.0070); CLR yielded +0.0063 (95% bootstrap CI −0.0032 to +0.0255). All percentile confidence intervals from 2000 common bootstrap resamples included zero. After bootstrap optimism correction, eight of the thirteen composites had negative ΔC estimates (HALP, modified HALP, PNI, SII, SIRI, PIV, PLR and MLR). Likelihood-ratio tests were nominally significant for 10 of the 15 additions, including eight of the thirteen composites, showing again that improved model fit did not necessarily translate into better patient ranking. Apparent calibration was broadly concordant across risk fifths for both models: across fifths of predicted risk, predicted five-year mortality ranged from 3.3% to 38.8% against an observed 2.9% to 38.5% for the clinical model, and from 2.9% to 40.7% against 1.0% to 42.3% once CRP and albumin were added.

Eight of the thirteen composites had adjusted hazard-ratio confidence intervals that excluded 1, whereas every confidence interval for ΔC over the clinical model included 0 (Figure 2).

### 3.6. Each Index Against Its Own Constituents

Every index in the panel is, by construction, a fixed-weight combination of its own constituents. For the twelve multiplicative indices the logarithm of the index is a fixed linear combination of the logarithms of its components, and PNI is a fixed linear combination of albumin and the lymphocyte count on the raw scale. It follows that no index can be entered into a model that already contains its own components: every such model failed for exact collinearity. A composite of this kind cannot add information to its constituents. What it can do is impose weights on them, and the empirical question is whether the imposed weights are as good as weights estimated from the data.

Because the model containing the composite is a restricted version of the model containing its components, the two are nested and can be compared directly (Table 5). The comparison was performed in maximal marker-specific samples (*n* = 518–606; 84–92 deaths) rather than requiring the complete 13-marker laboratory panel. Apparent differences in C-index, defined as freely weighted components minus the fixed index, were small (−0.0013 to +0.0057). Four nominal likelihood-ratio tests favoured freely estimated constituent weights in-sample: modified HALP (χ^2^ = 11.32 on 3 degrees of freedom, *p* = 0.010), CAR (χ^2^ = 3.94 on 1 degree of freedom, *p* = 0.047), PNI (χ^2^ = 4.29 on 1 degree of freedom, *p* = 0.038) and NPAR (χ^2^ = 10.20 on 2 degrees of freedom, *p* = 0.006). These *p* values are exploratory and were not adjusted for the thirteen comparisons. Internal validation gave similarly small but directionally inconsistent differences: across five-repeat five-fold cross-validation, ΔC ranged from −0.0089 to +0.0053. Paired 2000-resample bootstrap intervals around the cross-validated differences included zero for twelve of thirteen indices. MLR was the only index whose paired interval did not include zero, with a small difference favouring the fixed ratio (ΔC −0.0038, 95% CI −0.0070 to −0.0007). Because the bootstrap resampled the generated cross-validated prediction vectors rather than repeating the full cross-validation/model-fitting procedure within every resample, this isolated result is exploratory and should not be interpreted as definitive evidence of superior out-of-sample performance. It does not imply that MLR contains information absent from its components. Thus, the structural information-containment result and the empirical comparison of weighting schemes should be interpreted separately.

### 3.7. A Common Reference Model Containing CRP and Albumin

Adding CRP and albumin to the clinical model as two separate terms raised the C-index from 0.7720 to 0.7826 (ΔC +0.0106); both retained independent associations in that model (CRP adjusted hazard ratio 1.33, *p* = 0.008; albumin 1.23, *p* = 0.014). Against this reference model, none of the estimable composite indices showed evidence of further association or incremental discrimination (Table 6). Except for modified HALP (1.04 to 0.92), every adjusted hazard ratio moved towards unity, and every *p* value exceeded 0.19: CALLY 0.86 (*p* = 0.667), HALP 1.14 (*p* = 0.339), SII 1.17 (*p* = 0.197), NPAR 1.13 (*p* = 0.418), PNI 1.00 (*p* = 0.987), NLR 1.09 (*p* = 0.485), modified HALP 0.92 (*p* = 0.495). Apparent changes in the C-index were between −0.0013 and +0.0010, and every 1000-resample full-refit paired bootstrap 95% confidence interval included zero; across the twelve estimable composites the interval limits ranged from −0.0071 to +0.0099. The lymphocyte count, which appears in eleven of the thirteen indices, likewise showed no evidence of an adjusted association once CRP and albumin were in the model (adjusted hazard ratio 0.95, *p* = 0.667).

CAR could not be entered into this model at all. On the logarithmic scale used throughout, log(CAR) equals log(CRP) minus log(albumin); once both components are present the ratio is a perfect linear combination of them and carries, by construction, no additional information. The index with the strongest apparent performance in Table 3 is thus revealed to be a reparameterisation of its own constituents rather than a distinct marker.

The same argument applies to CLR with an additional consequence. Since log(CLR) equals log(CRP) minus log(lymphocyte count), CLR cannot be entered into a model already containing those two terms; and in a model containing the clinical covariates plus CRP and the lymphocyte count, the lymphocyte count itself showed no evidence of an adjusted association (adjusted hazard ratio 0.96, *p* = 0.755), so CLR carried little beyond CRP. More strikingly, CLR and CALLY returned identical estimates over the reference model (ΔC +0.0008, adjusted hazard ratio 0.86, *p* = 0.667) and could not be entered together. This is an algebraic identity rather than a coincidence: log(CALLY) equals log(albumin) plus log(lymphocyte count) minus log(CRP) minus a constant, so once albumin is in the model CALLY contributes only through log(CRP) minus log(lymphocyte count), which is log(CLR). Once albumin is in the model, CALLY and CLR become equivalent parameterisations of the same information, up to scaling and sign; two indices published under different names and with different stated rationales are therefore not independent markers.

### 3.8. Exploratory Analysis of a Data-Derived CLR Threshold

**Figure 3:**
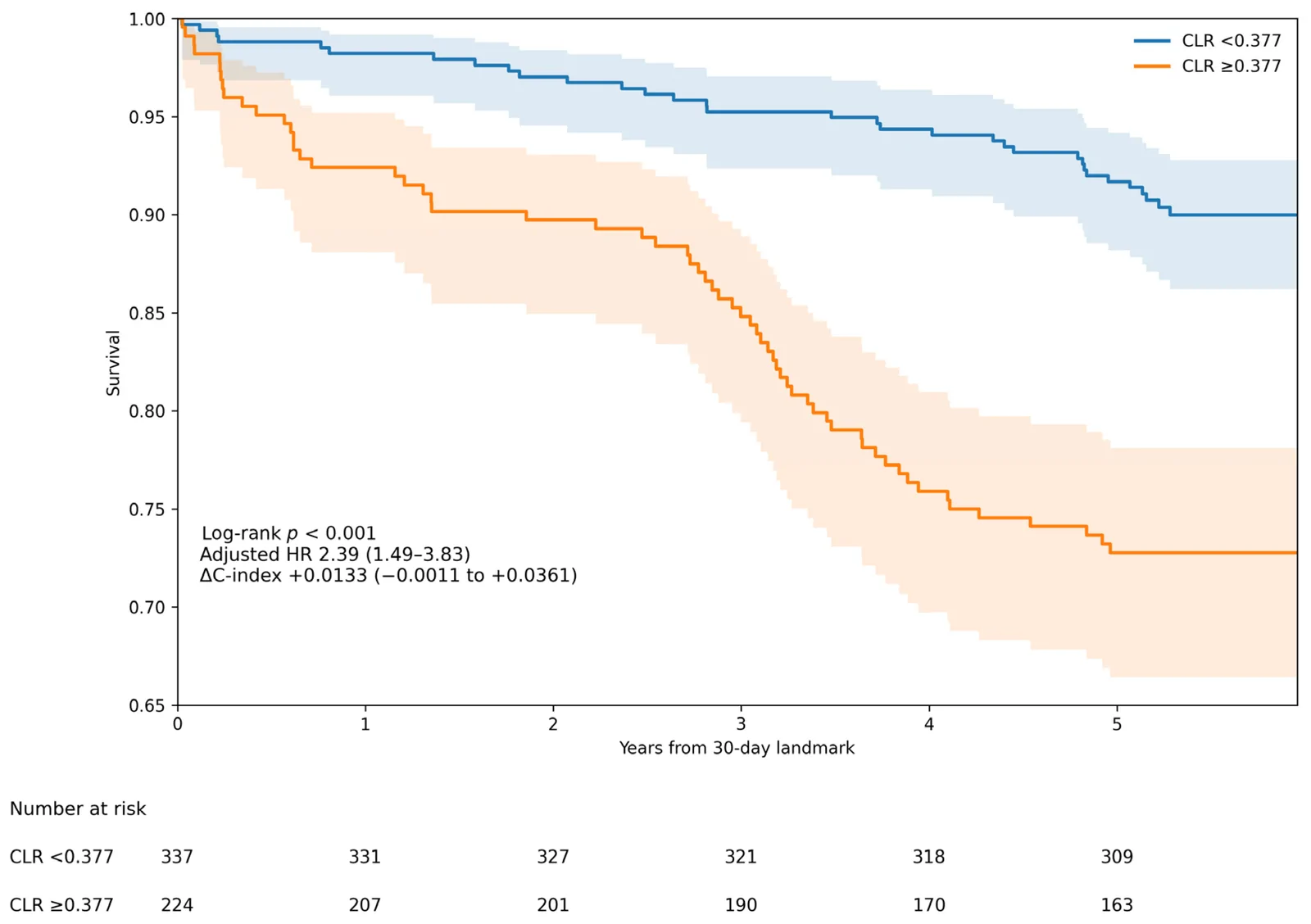
Exploratory Kaplan–Meier conditional survival among 30-day survivors according to the Youden-derived CLR threshold of 0.377. Time zero is the 30-day landmark, and the *x*-axis shows years from that landmark. The adjusted hazard ratio is 2.39 (95% CI 1.49–3.83), whereas the same dichotomy increases the C-index of the clinical model by 0.0133 (95% CI −0.0011 to +0.0361) and by 0.0037 once CRP and albumin are included. Shaded areas are 95% confidence intervals.

Because indices of this kind are frequently presented as dichotomies, we repeated the analysis for CLR using the Youden-derived threshold of 0.377. Mortality was 27.2% above the threshold and 9.8% below it, survival curves separated clearly (log-rank *p* < 0.001, Figure 3; Supplementary Table S6), the unadjusted hazard ratio was 3.22 and the adjusted hazard ratio was 2.39 (95% CI 1.49–3.83, *p* < 0.001). These are the numbers such analyses typically report. The corresponding gain in discrimination over the clinical model was +0.0133 (95% CI −0.0011 to +0.0361), and over the clinical model containing CRP and albumin it was +0.0037. A threshold chosen to maximise separation in the same dataset therefore produces an impressive hazard ratio and a survival figure that looks decisive, while adding almost nothing to the ability to rank patients by risk.

### 3.9. Sensitivity Analyses

The findings were stable in the primary analysis, the four prespecified sensitivity analyses and the additional admission-oriented sensitivity model, each applied to all twelve estimable indices (Supplementary Table S4). Using the fixed five-year endpoint, the increment attributable to CRP and albumin was +0.0156 and the composite *p* values ranged from 0.27 to 0.97. Retaining duplicate records gave +0.0145 with *p* values of 0.19 to 0.84, and excluding patients with non-obstructive coronary arteries (92 of the 561 analysed) gave +0.0102 with *p* values of 0.16 to 0.99. Omitting revascularisation from the clinical model lowered the clinical C-index to 0.7630 and raised the increment attributable to CRP and albumin to +0.0133; against that reference model the composite indices showed no statistical evidence of association (*p* 0.18 to 0.87) and their changes in the C-index remained between −0.0010 and +0.0018. In the additional admission-oriented model omitting both multivessel disease and revascularisation, the clinical C-index was 0.7603; CRP plus albumin increased it by +0.0152, composite *p* values ranged from 0.19 to 0.86, and composite ΔC values over that reference ranged from −0.0024 to +0.0019. In the post hoc model in which revascularisation strategy replaced any revascularisation, the clinical C-index was 0.7728 and the same eight composites remained nominally associated (hazard ratios 1.25–1.37); every 2000-resample bootstrap 95% confidence interval for ΔC over this clinical model included zero, and over the corresponding CRP-plus-albumin reference (ΔC +0.0108) composite *p* values ranged from 0.19 to 0.98. In no sensitivity analysis did any composite show nominal statistical evidence of association over the CRP-plus-albumin reference model. In the marker-specific available-case association analysis, sample sizes ranged from 518 to 606 with 84 to 92 deaths; the same eight composites that were nominally associated in the primary adjusted analysis remained nominally associated, while SIRI remained non-significant (*p* = 0.051) (Supplementary Table S2). The marker/reference-specific available-case decomposition directly addressing the CRP-plus-albumin comparison included 518 to 522 patients with 84 deaths; apparent ΔC values ranged from −0.0014 to +0.0011 and every estimable composite again had *p* ≥ 0.196 (Supplementary Table S3). Thus, neither the principal association pattern nor the common reference decomposition was materially changed by excluding patients solely because an unrelated laboratory measurement was unavailable.

## 4. Discussion

In 30-day survivors of NSTEMI with completely observed five-year vital status, we examined thirteen composite haematological and nutritional indices alongside their constituent measurements. We asked four questions of each index, and the answers are summarised in Table 7. Discrimination for long-term mortality was modest and closely clustered, with areas under the curve between 0.62 and 0.69. Eight indices were nominally associated with mortality after adjustment for the clinical model, yet none showed evidence of improved discrimination over that model. Against freely weighted models of their own constituents, fixed indices performed similarly: twelve of thirteen paired bootstrap intervals included zero, and the one exception, MLR, favoured the fixed ratio by a small margin and is best regarded as exploratory. This pattern separates information content from weighting: a deterministic composite cannot create information absent from its components, although imposed weights can sometimes reduce estimation variance. Once CRP and albumin were entered separately, no estimable composite showed statistical evidence of association with mortality, and CAR could not be estimated because it is an exact algebraic function of those two components.

The heavier clinical burden of the patients who died (Table 1) was largely captured by the clinical model (C-index 0.7720), leaving little room for any laboratory marker to improve discrimination. Replacing any revascularisation with revascularisation strategy in the clinical model did not change the findings (Supplementary Table S4).

These analyses answer related but distinct questions. The own-constituent comparison is the general structural test because it evaluates each index against the measurements from which that index is constructed. By contrast, the CRP-plus-albumin model is an empirical common benchmark. In this cohort, CRP and albumin accounted empirically for much of the prognostic signal across the panel, including blood-count-only indices; for SII, SIRI, PIV, NLR, PLR and MLR, however, this is a competing-biomarker comparison rather than an algebraic decomposition because neither CRP nor albumin is a constituent. The general inference therefore rests primarily on the own-constituent analysis, with the common reference analysis providing complementary evidence.

CALLY and CLR became equivalent parameterisations conditional on albumin. Although their raw values are not the same variable, once albumin is included their logarithmic contributions differ only by scaling and sign, and they returned identical adjusted estimates over the reference model. Their time-dependent areas under the curve differed by no more than 0.004 at any horizon. This illustrates that differently named composite indices can encode highly overlapping information. The platelet count itself provided no evidence of useful univariable discrimination in this cohort (AUC 0.486, 95% CI 0.418–0.554). In this dataset, imposing platelet count as either a divisor or multiplier in the two HALP variants did not generate demonstrable incremental prognostic value; the modified HALP score was the weakest index and showed no independent association with mortality (adjusted hazard ratio 1.04, *p* = 0.779).

Our findings do not contradict the primary results of previous studies so much as reinterpret them. Reports that the HALP score predicts in-hospital and one-year mortality in NSTEMI [5,7], that the CALLY index predicts mortality in acute coronary syndromes and after percutaneous coronary intervention [9,10,11], and that SII predicts long-term mortality after NSTEMI [13] all describe associations that we reproduce here. The larger 1648-patient NSTEMI analysis by Ilis et al. reported strong in-hospital discrimination for HALP [7], whereas HALP discrimination for five-year mortality was modest in our cohort; this difference is consistent with evidence that the prognostic performance of inflammatory indices varies with the time horizon considered [17]. Recent work in older patients with acute coronary syndrome likewise found CALLY independently associated with mortality but explicitly noted that its incremental value over established risk models and conventional biomarkers had not been formally demonstrated [11]. Prior NSTEMI work has also performed direct head-to-head comparisons: Karaca and Gumusdag compared NPAR with albumin and neutrophils individually, as well as with other inflammatory indices [16]. This represents a partial constituent comparison, but it did not test NPAR against a joint model containing its own constituents with freely estimated coefficients or quantify incremental discrimination over a clinical model. More recently, Zhang and Tang compared CALLY with CRP, albumin and lymphocyte count entered jointly in rheumatoid arthritis and found closely similar discrimination without significant differences in IDI or continuous NRI [18]. That study does not duplicate the present NSTEMI design, but it independently supports the distinction between comparison with single components and comparison with all components jointly. A comparable observation has also been reported in critically ill children, where CAR and CALLY discriminated mortality but reclassification analyses did not support incremental value beyond CRP alone [23]. In contrast, Buonpane et al. reported that the platelet-to-lymphocyte ratio/albumin ratio was independently associated with one-year mortality in 288 patients undergoing transcatheter aortic valve implantation, more strongly than either component alone, with an AUC of 0.69 at a data-derived cut-off [24]. That study differed from ours in population, outcome horizon and comparator: outperforming each component separately is a different question from outperforming a model that contains all constituents jointly. Read together, these studies indicate that the apparent value of a composite index depends on the clinical population, the outcome and, in particular, the comparator against which it is judged.

The threshold analysis makes the same point in the form in which readers most often encounter it. A hazard ratio of 2.39 with a clearly separated pair of survival curves is a persuasive display, yet the same dichotomy improved discrimination by 0.013 with a confidence interval crossing zero, and by 0.004 once CRP and albumin were in the model. Since the cut-point was chosen to maximise separation within the same dataset, both the hazard ratio and the figure are optimistic. Dichotomisation at a data-derived threshold should be reported as an exploratory secondary analysis, accompanied by the continuous estimate and a measure of discrimination. There is also a methodological point of wider relevance. Statistical significance in a multivariable model answers the question of whether a marker is associated with outcome after accounting for the covariates included; it does not answer whether the marker improves prediction, and it certainly does not answer whether the marker improves on a simpler version of itself. In this cohort, those three questions had different answers for the same variables. Any composite index whose constituents are themselves measured should therefore be reported alongside a model containing those constituents, and preferably with a measure of incremental discrimination in addition to the hazard ratio. Where a composite is a deterministic transformation of its components, as CAR is, the comparison is not merely advisable but definitional.

In 30-day survivors of NSTEMI whose routine admission panel already includes CRP, albumin and a complete blood count, as it did in our setting, the data do not support adding a composite index to risk assessment based on the clinical profile; combining measurements that are already reported together offers little convenience. When a composite value is abnormal, the components should be read directly: a low HALP score may reflect anaemia, hypoalbuminaemia, lymphopenia or thrombocytosis, and a low CALLY index may also reflect systemic inflammation, each with different clinical implications. In this cohort, CRP and albumin carried much of the measurable laboratory signal, although the data do not support replacing an established risk score with these two measurements. Cut-offs derived from a single dataset, such as the CLR threshold examined here, should not guide individual decisions without external validation. These implications concern risk discrimination; whether any marker should change management was not tested. Because the benchmark was a study-specific clinical model rather than an established score, the findings should not be extrapolated to incremental performance over GRACE or other validated NSTEMI risk models.

**Figure 4:**
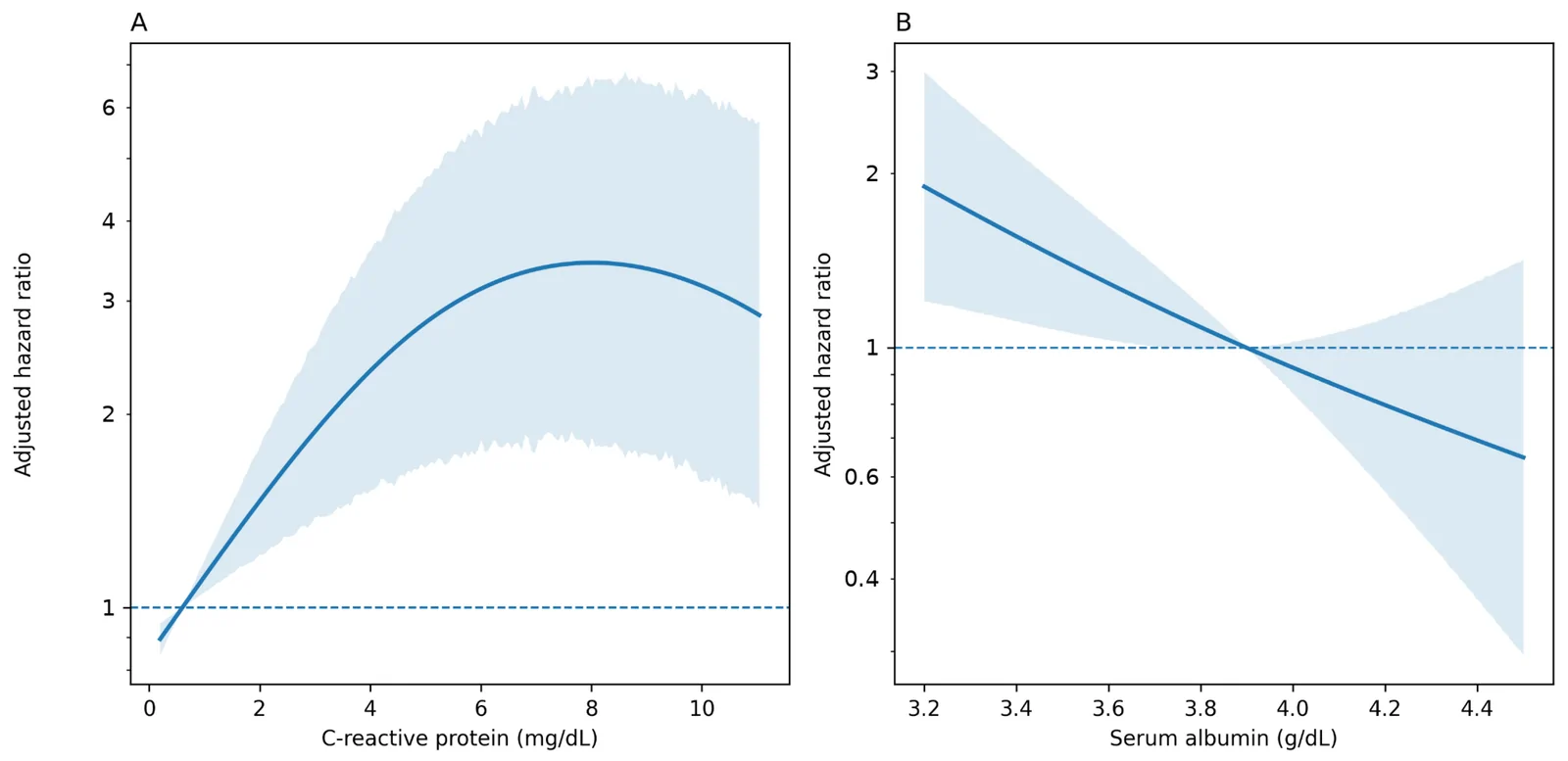
Adjusted association of C-reactive protein and albumin, the two laboratory terms in the common reference model, modelled with restricted cubic splines and referenced to the cohort median. Splines were fitted on the logarithmic scale, on which all models are specified, and hazard ratios, shown on a logarithmic axis, are plotted against untransformed values. (**A**) C-reactive protein (non-linearity *p* = 0.552; overall adjusted association *p* = 0.029). (**B**) Serum albumin (non-linearity *p* = 0.820; overall *p* = 0.096). Shaded areas are 95% confidence intervals. Solid lines show the adjusted hazard ratio, and dashed horizontal lines mark a hazard ratio of 1.

Our results should not be read as showing that inflammatory and nutritional status is prognostically unimportant after NSTEMI. CRP and albumin were independently associated with mortality, and their joint addition to the clinical model produced the largest increment observed in this study. Inflammatory ratios have also been linked to coronary microvascular dysfunction: in chronic coronary syndrome without obstructive stenosis, NLR and PLR were higher in patients with impaired microcirculatory function, and PLR was associated with adverse outcome [25]. The CRP spline was compatible with a broadly graded association across most of the observed range, but uncertainty widened substantially at the high-CRP tail (Figure 4A). The point is narrower: in the common reference analysis, the increment was obtained from the two raw measurements, and no estimable composite demonstrated incremental discrimination when added to that benchmark. This empirical finding should be read alongside, rather than as a substitute for, the own-constituent analysis.

The 125 patients excluded for an incomplete laboratory panel had about half the mortality of those analysed (8.0% versus 16.8%; Supplementary Table S1), so the analytic cohort was enriched for events, and absolute risks and areas under the curve from this cohort may not transfer to an unselected NSTEMI population. The central comparisons, however, are relative and made within the same patients: each composite against its own constituents, or against a common reference model. When the own-constituent comparison was performed in maximal marker-specific samples (*n* = 518–606) and the association and CRP-plus-albumin analyses were repeated in available-case samples, the pattern was unchanged (Table 5; Supplementary Tables S2 and S3).

### Limitations

Several features of the study constrain the interpretation of these findings. The retrospective, single-centre design limits generalisability and leaves the possibility of residual confounding. The cohort was comparatively young (mean age 59 years), predominantly male and largely uncomplicated (Killip class ≥ 2 in 3.6%, mean estimated glomerular filtration rate 85 mL/min/1.73 m^2^), whereas these indices are most often proposed for older and higher-risk populations. The clinical model was assembled from variables routinely available in the registry rather than taken from a validated risk score, and whether any index adds to an external instrument such as GRACE could not be addressed because its components were not all recorded. Although the event count was sufficient for the prespecified clinical model, it provides limited precision for estimating small changes in discrimination, as reflected by the relatively wide confidence intervals around ΔC. Our inference therefore depends less on any individual null comparison than on the consistent absence of incremental discrimination across the marker panel. Laboratory measurements were obtained only at admission, during an evolving acute-phase response, and the prognostic behaviour of serial measurements may differ. All-cause mortality was used because cause-specific death was not adjudicated. Completeness of revascularisation at the lesion level was not recorded, so residual confounding by treatment strategy cannot be excluded. Because the analysis was landmarked at 30 days, the findings apply to 30-day survivors and should not be extrapolated to early mortality after NSTEMI. The complete-panel requirement excluded 125 otherwise eligible patients whose mortality was lower than that of the analysed cohort, as discussed above; common-sample ROC and Cox analyses remain subject to this selection. Although thirteen indices were evaluated, the panel was not exhaustive; indices requiring measurements unavailable in the registry, including fibrinogen and red cell distribution width (the latter available in only 67% of patients), could not be assessed. Proportional-hazards testing was focused on the common clinical-plus-CRP-and-albumin reference model, and detailed functional-form testing was limited to CRP and albumin; marker-specific assumption checks were therefore not exhaustive. Finally, the present analyses address discrimination and do not rule out other potential applications of composite indices, such as calibration within selected risk strata or longitudinal monitoring.

## 5. Conclusions

Among 30-day survivors of NSTEMI followed for a median of 5.48 years, thirteen composite haematological and nutritional indices showed modest and closely similar discrimination for all-cause mortality. Although several were nominally associated with mortality after adjustment for the prespecified study clinical model, none showed evidence of incremental discrimination over that model, and no estimable composite showed statistical evidence of association once CRP and albumin were entered as separate terms in the empirical common reference model; bootstrap confidence intervals for the corresponding ΔC estimates all included zero. Deterministic composites contained no information unavailable in their own measured constituents. In marker-specific internal validation, fixed-versus-freely weighted constituent differences were small and inconsistent; MLR showed one isolated small difference favouring the fixed ratio, but the corresponding interval was conditional on the generated cross-validated predictions and is exploratory rather than definitive. CALLY and CLR became equivalent parameterisations conditional on albumin rather than representing identical raw variables. With 84 events in the common-sample multivariable analysis, the confidence intervals around the change in the C-index over the clinical model do not exclude increments of up to approximately 0.03, so these are null findings rather than demonstrations of equivalence. Much of the observed composite-marker signal in this cohort was already present in constituent measurements. These results apply to patients who survived the first 30 days and do not address incremental value over validated risk scores such as GRACE.

## Figures and Tables

**Table 1:** Baseline characteristics according to five-year survival.

Variable	All (*n* = 561)	Survivors (*n* = 475)	Deaths (*n* = 86)	*p*
Age, years	59.4 ± 10.7	58.6 ± 10.4	63.8 ± 11.0	<0.001
Male sex	433 (77.2)	369 (77.7)	64 (74.4)	0.600
Hypertension	310 (55.3)	248 (52.2)	62 (72.1)	<0.001
Diabetes mellitus	174 (31.0)	136 (28.6)	38 (44.2)	0.006
Current smoking	256 (45.6)	223 (46.9)	33 (38.4)	0.177
Prior myocardial infarction	133 (23.7)	103 (21.7)	30 (34.9)	0.012
Prior stroke	18 (3.2)	12 (2.5)	6 (7.0)	0.043
Systolic blood pressure, mmHg	149.7 ± 28.6	149.0 ± 28.8	153.8 ± 27.3	0.149
Heart rate, beats/min	84.1 ± 23.2	82.9 ± 23.0	90.4 ± 23.2	0.008
Killip class ≥ 2	20 (3.6)	15 (3.2)	5 (5.8)	0.212
LVEF, %	51.7 ± 9.7	52.8 ± 8.9	45.3 ± 11.7	<0.001
eGFR, mL/min/1.73 m^2^	85.0 ± 20.6	87.5 ± 18.8	70.2 ± 24.6	<0.001
Non-obstructive coronary arteries	92 (16.4)	80 (16.8)	12 (14.0)	0.612
Multivessel disease	220 (39.2)	182 (38.3)	38 (44.2)	0.365
Percutaneous coronary intervention	298 (53.1)	259 (54.5)	39 (45.3)	0.147
Coronary artery bypass grafting	77 (13.7)	71 (14.9)	6 (7.0)	0.071
Any revascularisation	375 (66.8)	330 (69.5)	45 (52.3)	0.003
Multivessel revascularisation ^a^	107/220 (48.6)	99/182 (54.4)	8/38 (21.1)	<0.001
Peak troponin, ng/mL	0.78 (0.10–4.76)	0.70 (0.10–4.17)	1.84 (0.17–11.40)	0.070
Admission glucose, mg/dL	116 (99–168)	113 (98–158)	128 (108–214)	0.001
Components				
C-reactive protein, mg/dL	0.60 (0.30–1.60)	0.60 (0.30–1.30)	1.35 (0.53–4.78)	<0.001
Albumin, g/dL	3.9 ± 0.3	3.9 ± 0.3	3.7 ± 0.3	<0.001
Haemoglobin, g/dL	14.1 ± 1.8	14.3 ± 1.8	13.3 ± 2.0	<0.001
Lymphocytes, ×10^9^/L	2.5 ± 1.1	2.5 ± 1.1	2.2 ± 1.1	0.004
Neutrophils, ×10^9^/L	6.7 ± 3.1	6.5 ± 2.9	7.6 ± 3.9	0.019
Platelets, ×10^9^/L	240.2 ± 67.4	239.9 ± 64.4	241.8 ± 82.5	0.838
Composite indices				
HALP	60.1 ± 30.7	62.0 ± 30.4	49.2 ± 30.5	<0.001
Modified HALP, ×10^6^	2.93 (1.89–4.26)	3.07 (1.97–4.39)	2.43 (1.50–3.67)	<0.001
CALLY	1.42 (0.51–3.16)	1.63 (0.66–3.30)	0.48 (0.19–1.73)	<0.001
CAR	0.16 (0.07–0.41)	0.14 (0.07–0.35)	0.36 (0.13–1.17)	<0.001
CLR	0.27 (0.12–0.77)	0.24 (0.12–0.60)	0.77 (0.22–1.95)	<0.001
PNI	51.0 ± 6.5	51.6 ± 6.2	48.2 ± 7.2	<0.001
NPAR	16.5 (14.4–19.3)	16.3 (14.3–19.0)	18.5 (16.3–20.8)	<0.001
SII	545 (397–950)	514 (383–890)	706 (534–1155)	<0.001
SIRI	1.36 (0.89–2.33)	1.29 (0.86–2.08)	1.87 (1.04–3.45)	<0.001
PIV	315 (199–539)	300 (196–509)	432 (265–785)	<0.001
NLR	2.42 (1.75–3.80)	2.29 (1.69–3.60)	3.24 (2.17–5.13)	<0.001
PLR	96 (74–135)	94 (71–130)	112 (88–174)	<0.001
MLR	0.24 (0.18–0.33)	0.24 (0.18–0.32)	0.31 (0.21–0.48)	<0.001

Values are mean ± standard deviation, median (interquartile range) or *n* (%). LVEF, left ventricular ejection fraction; eGFR, estimated glomerular filtration rate; HALP, haemoglobin–albumin–lymphocyte–platelet score; CALLY, C-reactive protein–albumin–lymphocyte index; CAR, C-reactive protein-to-albumin ratio; PNI, prognostic nutritional index; NPAR, neutrophil percentage-to-albumin ratio; SII, systemic immune-inflammation index; SIRI, systemic inflammation response index; PIV, pan-immune-inflammation value; NLR, neutrophil-to-lymphocyte ratio; PLR, platelet-to-lymphocyte ratio; MLR, monocyte-to-lymphocyte ratio. Categorical variables were compared with the chi-square test, or with Fisher’s exact test when an expected cell count was below five (prior stroke, Killip class ≥ 2). ^a^ Among patients with multivessel disease: coronary artery bypass grafting, or percutaneous coronary intervention of more than one vessel at the index procedure or as a staged procedure; angiographic completeness of revascularisation was not recorded.

**Table 2:** Discrimination for five-year all-cause mortality and comparison with C-reactive protein.

Marker	AUC	95% CI	ΔAUC vs. CRP	DeLong *p*
CALLY	0.689	0.623–0.756	+0.023	0.035
CLR	0.685	0.618–0.751	+0.018	0.080
CAR	0.674	0.607–0.740	+0.007	0.043
CRP	0.666	0.600–0.733	reference	–
NPAR	0.661	0.599–0.723	−0.006	0.883
NLR	0.651	0.591–0.712	−0.015	0.699
PNI	0.647	0.579–0.716	−0.019	0.674
HALP	0.644	0.579–0.708	−0.023	0.576
Haemoglobin	0.641	0.576–0.707	−0.025	0.516
SII	0.640	0.579–0.701	−0.027	0.484
Albumin	0.635	0.566–0.703	−0.032	0.469
SIRI	0.628	0.560–0.696	−0.039	0.323
MLR	0.621	0.550–0.692	−0.045	0.265
Lymphocytes	0.620	0.556–0.684	−0.046	0.292
Modified HALP	0.619	0.553–0.685	−0.048	0.288
PLR	0.618	0.556–0.681	−0.048	0.245
PIV	0.616	0.549–0.683	−0.050	0.194
Neutrophils	0.597	0.532–0.662	−0.069	0.084
Platelets	0.486	0.418–0.554	−0.180	<0.001

Index definitions: HALP = haemoglobin (g/L) × albumin (g/L) × lymphocytes (×10^9^/L)/platelets (×10^9^/L); modified HALP = haemoglobin (g/L) × albumin (g/L) × lymphocytes (×10^9^/L) × platelets (×10^9^/L); CALLY = albumin (g/dL) × lymphocytes (×10^9^/L)/(CRP (mg/dL) × 10); CAR = CRP/albumin; CLR = CRP/lymphocytes; PNI = 10 × albumin (g/dL) + 5 × lymphocytes (×10^9^/L); NPAR = neutrophil percentage/albumin (g/dL); SII = platelets × neutrophils/lymphocytes; SIRI = neutrophils × monocytes/lymphocytes; PIV = neutrophils × platelets × monocytes/lymphocytes; NLR, PLR and MLR are the corresponding ratios. All markers were oriented so that higher values denote higher risk before analysis. Eighteen pairwise comparisons were made against CRP; *p* values are unadjusted and a Bonferroni-corrected threshold would be 0.0028. AUC, area under the receiver operating characteristic curve.

**Table 3:** Cox proportional-hazards models for all-cause mortality.

Marker	Unadjusted HR	*p*	Adjusted HR (95% CI)	*p*
CAR	1.65	<0.001	1.38 (1.12–1.69)	0.003
NPAR	1.59	<0.001	1.36 (1.07–1.73)	0.012
CRP	1.62	<0.001	1.35 (1.10–1.66)	0.005
CALLY	1.68	<0.001	1.34 (1.09–1.65)	0.006
CLR	1.65	<0.001	1.32 (1.07–1.62)	0.010
Neutrophils	1.36	0.002	1.32 (1.06–1.64)	0.014
SII	1.38	<0.001	1.29 (1.03–1.61)	0.026
HALP	1.44	<0.001	1.29 (1.02–1.64)	0.036
PNI	1.53	<0.001	1.29 (1.01–1.65)	0.040
Haemoglobin	1.47	<0.001	1.28 (1.03–1.60)	0.029
Albumin	1.34	<0.001	1.27 (1.07–1.51)	0.007
PIV	1.32	0.002	1.26 (1.01–1.57)	0.037
SIRI	1.34	0.001	1.22 (0.97–1.53)	0.088
NLR	1.39	<0.001	1.21 (0.97–1.52)	0.092
Platelets	1.01	0.914	1.20 (0.97–1.48)	0.093
PLR	1.27	0.008	1.16 (0.92–1.46)	0.213
MLR	1.24	0.019	1.09 (0.86–1.39)	0.466
Modified HALP	1.40	<0.001	1.04 (0.81–1.32)	0.779
Lymphocytes	1.28	0.006	1.04 (0.81–1.33)	0.759

Hazard ratios are per one standard deviation adverse change on the logarithmic scale. Adjusted models (*n* = 518, 84 deaths) include age, male sex, diabetes mellitus, hypertension, prior myocardial infarction, left ventricular ejection fraction, estimated glomerular filtration rate, Killip class ≥ 2, multivessel disease and revascularisation. Marker-wise *p* values are nominal and were not adjusted for multiplicity. The reported Schoenfeld residual test pertains to the common clinical-plus-CRP-and-albumin reference model (minimum covariate *p* = 0.116; global χ^2^ = 8.88, *p* = 0.713), not to every marker-specific model.

**Table 4:** Incremental discrimination over the clinical model (C-index 0.7720).

Marker Added	C-Index	ΔC	Bootstrap 95% CI	Optimism-Corrected ΔC	LR χ^2^	*p*
CAR	0.7805	+0.0085	−0.0030 to +0.0287	+0.0076	8.76	0.003
CRP	0.7794	+0.0073	−0.0030 to +0.0280	+0.0062	7.86	0.005
CALLY	0.7791	+0.0070	−0.0036 to +0.0257	+0.0059	7.31	0.007
CLR	0.7784	+0.0063	−0.0032 to +0.0255	+0.0052	6.58	0.010
Albumin	0.7752	+0.0032	−0.0042 to +0.0184	+0.0031	5.95	0.015
NPAR	0.7752	+0.0031	−0.0051 to +0.0185	+0.0009	6.06	0.014
NLR	0.7745	+0.0024	−0.0036 to +0.0141	+0.0007	2.76	0.096
Modified HALP	0.7725	+0.0004	−0.0043 to +0.0093	−0.0027	0.08	0.779
SII	0.7719	−0.0002	−0.0057 to +0.0134	−0.0015	4.78	0.029
HALP	0.7714	−0.0007	−0.0088 to +0.0096	−0.0018	4.27	0.039
PLR	0.7709	−0.0012	−0.0063 to +0.0072	−0.0031	1.52	0.218
PNI	0.7707	−0.0014	−0.0091 to +0.0121	−0.0035	3.98	0.046
MLR	0.7706	−0.0015	−0.0075 to +0.0108	−0.0062	0.52	0.470
SIRI	0.7705	−0.0016	−0.0072 to +0.0132	−0.0055	2.79	0.095
PIV	0.7681	−0.0040	−0.0093 to +0.0129	−0.0077	4.10	0.043

Bootstrap percentile confidence intervals and Harrell optimism correction, both from 2000 resamples drawn in common across all thirteen composite indices plus CRP and albumin. No confidence interval excludes zero. The likelihood-ratio test on 1 degree of freedom is reported for the same addition; it is nominally significant for 10 of the 15 additions, including eight of the thirteen composites. The two columns answer different questions: the likelihood-ratio test asks whether model fit improves, whereas the change in the C-index asks whether patients are ranked better. Values shown are from an independent statsmodels PHReg reproduction and agree closely with the primary lifelines implementation.

**Table 5:** Marker-specific comparison of each composite index with a freely weighted model of its own constituents.

Index	*n*	Deaths	Apparent ΔC	5 × 5-fold CV ΔC (95% CI)	LR χ^2^	*p*
HALP	553	87	+0.0045	+0.0018 (−0.0108 to +0.0143)	7.23	0.065
Modified HALP	553	87	+0.0020	+0.0053 (−0.0128 to +0.0226)	11.32	0.010
CALLY	518	84	+0.0045	+0.0036 (−0.0092 to +0.0154)	5.72	0.057
CAR	551	84	+0.0035	+0.0010 (−0.0125 to +0.0136)	3.94	0.047
CLR	569	89	+0.0006	−0.0009 (−0.0090 to +0.0067)	1.38	0.240
PNI	553	87	+0.0056	+0.0024 (−0.0116 to +0.0158)	4.29	0.038
NPAR	553	87	+0.0057	+0.0016 (−0.0164 to +0.0202)	10.20	0.006
SII	606	92	−0.0010	−0.0058 (−0.0149 to +0.0020)	2.45	0.294
SIRI	606	92	+0.0037	−0.0007 (−0.0096 to +0.0085)	1.86	0.395
PIV	606	92	+0.0025	−0.0089 (−0.0182 to +0.0002)	2.10	0.551
NLR	606	92	+0.0008	+0.0033 (−0.0067 to +0.0126)	2.54	0.111
PLR	606	92	−0.0013	−0.0024 (−0.0127 to +0.0065)	1.91	0.167
MLR	606	92	+0.0000	−0.0038 (−0.0070 to −0.0007)	0.01	0.918

Each row uses the maximal sample with the clinical covariates, the relevant composite and all of its own constituents observed. Apparent ΔC and cross-validated ΔC are defined as C (+components) minus C (+index), so positive values favour freely weighted constituents and negative values favour the fixed composite. Internal validation used five repeats of stratified five-fold cross-validation; each patient’s out-of-fold linear predictor was averaged across repeats, and the 95% confidence interval is from a paired 2000-resample patient-level bootstrap of the two cross-validated prediction vectors. Because the bootstrap resamples the generated cross-validated prediction vectors rather than repeating the complete cross-validation/model-fitting procedure within every resample, this interval is conditional on those predictions and is interpreted descriptively. The likelihood-ratio test on k − 1 degrees of freedom compares the restricted fixed-weight index model with the freely weighted constituent model and is an in-sample model-fit test. PNI and its components are standardised on the raw scale because PNI is additive; the other multiplicative indices are compared on the logarithmic scale. The thirteen likelihood-ratio *p* values are nominal and exploratory and were not adjusted for multiplicity.

**Table 6:** Contribution of each composite index over a reference model containing the clinical model plus C-reactive protein and albumin as separate terms (C-index 0.7826).

Index Added	ΔC	Adjusted HR	*p*	ΔC 95% CI
MLR	+0.0010	0.92	0.531	−0.0048 to +0.0085
CALLY	+0.0008	0.86	0.667	−0.0043 to +0.0068
NPAR	+0.0008	1.13	0.418	−0.0039 to +0.0099
CLR	+0.0008	0.86	0.667	−0.0043 to +0.0068
SIRI	+0.0001	1.04	0.779	−0.0043 to +0.0084
NLR	+0.0000	1.09	0.485	−0.0038 to +0.0079
PNI	−0.0001	1.00	0.987	−0.0049 to +0.0059
Modified HALP	−0.0002	0.92	0.495	−0.0040 to +0.0071
SII	−0.0004	1.17	0.197	−0.0055 to +0.0082
PIV	−0.0006	1.10	0.444	−0.0057 to +0.0088
PLR	−0.0009	1.07	0.606	−0.0062 to +0.0062
HALP	−0.0013	1.14	0.339	−0.0071 to +0.0056
CAR	not estimable	–	–	–
Individual components				–
Neutrophils	+0.0020	1.22	0.094	–
Haemoglobin	+0.0007	1.22	0.085	–
Lymphocytes	+0.0008	0.95	0.667	–
Platelets	−0.0012	1.18	0.121	–

The reference model comprises the clinical model plus C-reactive protein and albumin entered separately (CRP HR 1.33, *p* = 0.008; albumin HR 1.23, *p* = 0.014; ΔC +0.0106 over the clinical model). For the twelve estimable composites, ΔC 95% confidence intervals are from a 1000-resample full-refit paired bootstrap using common resamples; all include zero. CAR is not estimable because log(CAR) = log(CRP) − log(albumin). CALLY/CLR equivalence conditional on albumin is detailed in the Results.

**Table 7:** Four questions asked of each composite index and the corresponding findings.

Question	Analysis	Finding
Is the index associated with mortality after clinical adjustment?	Cox regression adjusted for the clinical model (Table 3)	Eight of thirteen composites nominally associated (HR 1.26–1.38 per 1 SD adverse change)
Does it improve discrimination over the clinical model?	Change in Harrell’s C-index with 2000-resample bootstrap CI and optimism correction (Table 4)	No evidence of improvement; every 95% CI included zero, and eight of thirteen increments were negative after optimism correction
Does it carry information beyond its own constituents?	Nested comparison with a freely weighted model of its constituents; 5 × 5-fold cross-validation (Table 5)	No information beyond the constituents by construction; cross-validated ΔC −0.0089 to +0.0053; 12 of 13 intervals included zero (MLR exploratory, favouring the fixed ratio)
Does it add to CRP and albumin?	Clinical model plus CRP and albumin as separate terms (Table 6)	No evidence of association (all *p* ≥ 0.19) or improved discrimination for any estimable composite; CAR not estimable

HR, hazard ratio; SD, standard deviation; CI, confidence interval; ΔC, change in Harrell’s C-index; MLR, monocyte-to-lymphocyte ratio; CRP, C-reactive protein; CAR, C-reactive protein-to-albumin ratio.

## Data Availability

The source registry contains directly identifying information and cannot be shared. A de-identified dataset and the complete analysis script are available from the corresponding author on reasonable request and with institutional approval.

## Supplementary Materials

The following supporting information can be downloaded at: https://www.mdpi.com/article/10.3390/medicina62101965/s1, Supplementary Figure S1: Patient flow diagram; Supplementary Table S1: Comparison of analysed patients with patients excluded because of an incomplete laboratory panel; Supplementary Table S2: Marker-specific available-case association sensitivity analysis; Supplementary Table S3: Marker/reference-specific available-case CRP-plus-albumin decomposition; Supplementary Table S4: Sensitivity analyses; Supplementary Table S5: Discrimination for all-cause mortality at one, three and five years; Supplementary Table S6: Continuous versus dichotomised CLR.

## References

[1] Byrne R.A., Rossello X., Coughlan J.J., Barbato E., Berry C., Chieffo A., Claeys M.J., Dan G.-A., Dweck M.R., Galbraith M. (2023). 2023 ESC Guidelines for the management of acute coronary syndromes. Eur. Heart J..

[2] Polonski L., Gasior M., Gierlotka M., Osadnik T., Kalarus Z., Trusz-Gluza M., Zembala M., Wilczek K., Lekston A., Zdrojewski T. (2011). A comparison of ST elevation versus non-ST elevation myocardial infarction outcomes in a large registry database: Are non-ST myocardial infarctions associated with worse long-term prognoses?. Int. J. Cardiol..

[3] Moledina S.M., Weight N., Cole A., Rashid M., Kontopantelis E., Mamas M.A. (2025). The impact of specialist cardiology inpatient care on the long-term outcomes of non-ST-segment elevation myocardial infarction (NSTEMI): A nationwide cohort study. Int. J. Cardiol..

[4] Chen X.L., Xue L., Wang W., Chen H.N., Zhang W.H., Liu K., Chen X.Z., Yang K., Zhang B., Chen Z.X. (2015). Prognostic significance of the combination of preoperative hemoglobin, albumin, lymphocyte and platelet in patients with gastric carcinoma: A retrospective cohort study. Oncotarget.

[5] Kiliç R., Güzel T., Aktan A., Güzel H., Kaya A.F., Çankaya Y. (2025). The effectiveness of HALP score in predicting mortality in non-ST-elevation myocardial infarction patients. Coron. Artery Dis..

[6] Toprak K., Toprak İ.H., Acar O., Ermiş M.F. (2024). The predictive value of the HALP score for no-reflow phenomenon and short-term mortality in patients with ST-elevation myocardial infarction. Postgrad. Med..

[7] Ilis D., Arslan A., Artac I., Karakayali M., Omar T., Hamideyin S., Taşkan H., Yagicibulut O., Karabag Y., Rencuzogullari I. (2025). Prognostic value of HALP score in predicting in-hospital mortality in patients with NSTEMI. Biomark. Med..

[8] Kocaoğlu S., Alatlı T. (2022). The efficiency of the HALP score and the modified HALP score in predicting mortality in patients with acute heart failure presenting to the emergency department. J. Coll. Physicians Surg. Pak..

[9] Güven B., Deniz M.F., Geylan N.A., Kültürsay B., Dönmez A., Bulat Z., Gül Ö.B., Kaya M., Oktay V. (2025). A novel indicator of all-cause mortality in acute coronary syndrome: The CALLY index. Biomark. Med..

[10] Demir Y., Sevinc S. (2025). The prognostic value of C-reactive protein–albumin–lymphocyte (CALLY) index in predicting in-hospital mortality after primary percutaneous coronary intervention in patients with ST-elevation myocardial infarction (STEMI). Catheter. Cardiovasc. Interv..

[11] Serin E., Sarılar M., Şahin S., Er Kara A., Değirmenci H., Kalender E., Gürdal A., Keskin K. (2026). Is the Cally index truly a prognostic marker that predicts mortality in older patients with acute coronary syndrome?. Front. Cardiovasc. Med..

[12] Söğüt Ö., Akdemir T., Can M.M. (2021). Prognostic value of the C-reactive protein to albumin ratio in patients undergoing primary percutaneous coronary intervention for ST-segment elevation myocardial infarction. Turk. J. Med. Sci..

[13] Yaşan M., Özel R., Yildiz A., Savaş G., Korkmaz A. (2024). The predictive value of systemic immune-inflammation index for long-term cardiovascular mortality in non-ST segment elevation myocardial infarction. Coron. Artery Dis..

[14] Ozilhan M.O., Çakmak Karaaslan O., Acikgoz S.K., Selcuk H., Selcuk M.T., Maden O. (2023). Systemic inflammation response index is associated MACE in patients with NSTEMI. Eur. Rev. Med. Pharmacol. Sci..

[15] Bećirović E., Bećirović M., Šegalo S., Bećirović A., Hadžić S., Ljuca K., Papić E., Ferhatbegović L., Ejubović M., Jagodić Ejubović A. (2025). Hemogram-derived ratios as prognostic markers for major adverse cardiovascular events in patients with non-ST-segment elevation myocardial infarction. World J. Methodol..

[16] Karaca M., Gumusdag A. (2024). Prognostic role of neutrophil percentage-to-albumin ratio in patients with non-ST-elevation myocardial infarction. Medicina.

[17] Mitsis A., Tsiaktani K., Tasoulis S.K., Lakoumentas J., Karmioti G., Kyriakou M., Sokratous S., Drakomathioulakis M., Eftychiou C., Tzikas S. (2026). Time-dependent prognostic performance of inflammatory biomarkers after AMI: Results from the INFINITY study. Biomark. Med..

[18] Zhang T., Tang L.V. (2026). Association between CALLY index and all-cause mortality in patients with rheumatoid arthritis. Front. Nutr..

[19] Aslanger E.K., Yıldırımtürk Ö., Şimşek B., Bozbeyoğlu E., Şimşek M.A., Karabay C.Y., Smith S.W., Değertekin M. (2020). DIagnostic accuracy oF electrocardiogram for acute coronary OCClUsion resuLTing in myocardial infarction (DIFOCCULT Study). Int. J. Cardiol. Heart Vasc..

[20] Aslanger E., Yıldırımtürk Ö., Şimşek B., Sungur A., Türer Cabbar A., Bozbeyoğlu E., Karabay C.Y., Smith S.W., Değertekin M. (2020). A new electrocardiographic pattern indicating inferior myocardial infarction. J. Electrocardiol..

[21] DeLong E.R., DeLong D.M., Clarke-Pearson D.L. (1988). Comparing the areas under two or more correlated receiver operating characteristic curves: A nonparametric approach. Biometrics.

[22] Harrell F.E., Lee K.L., Mark D.B. (1996). Multivariable prognostic models: Issues in developing models, evaluating assumptions and adequacy, and measuring and reducing errors. Stat. Med..

[23] Atay G., Erdoğan S., Pençe İ., Pençe M.E., Kurt D. (2026). CRP-to-albumin ratio and CALLY index for sepsis identification and mortality discrimination in critically ill children. Pediatr. Int..

[24] Buonpane A., Romagnoli E., Bianchini F., Bianchini E., Marchetta M., Aurigemma C., Bruno P., Nesta M., Lunardi M., Leone A.M. (2026). Integrating inflammatory and nutritional markers: The prognostic value of the platelet-to-lymphocyte/albumin ratio for one-year all-cause mortality in patients undergoing transcatheter aortic valve implantation. Minerva Cardiol. Angiol..

[25] Szolc P., Niewiara Ł., Kawulak M., Szkodoń K., Ostrowska-Kaim E., Nosal M., Krzanowski M., Żmudka K., Legutko J., Guzik B. (2020). Neutrophil-lymphocyte ratio and platelet-lymphocyte ratio as predictors of coronary microcirculatory disease occurrence and outcome in patients with chronic coronary syndrome and no significant coronary artery stenosis. Wiad. Lek..

