# Supplementary material for: Composite Haematological and Nutritional Indices Show No Evidence of Improved Long-Term Mortality Discrimination Beyond Their Own Components in Non-ST-Segment Elevation Myocardial Infarction
Source: Medicina (Kaunas). 2026 Oct 11;62(10):1965. doi: 10.3390/medicina62101965 (PMC13706856; doi:10.3390/medicina62101965)
Supplement: Supplementary file 1 [file medicina-62-01965-s001.zip › medicina-4558738-supplementary.pdf]

Supplementary Materials

Composite haematological and nutritional indices show no evidence of improved long-term mortality discrimination beyond their own components in non-ST-segment elevation myocardial infarction

Emrah Bozbeyoğlu[1,2,*], Aylin Sungur[3], Fatma Can[1], Barış Şimşek[1], Duygu Genç Albayrak[2], Funda Özlem Pamuk[2], Can Yücel Karabay[4]

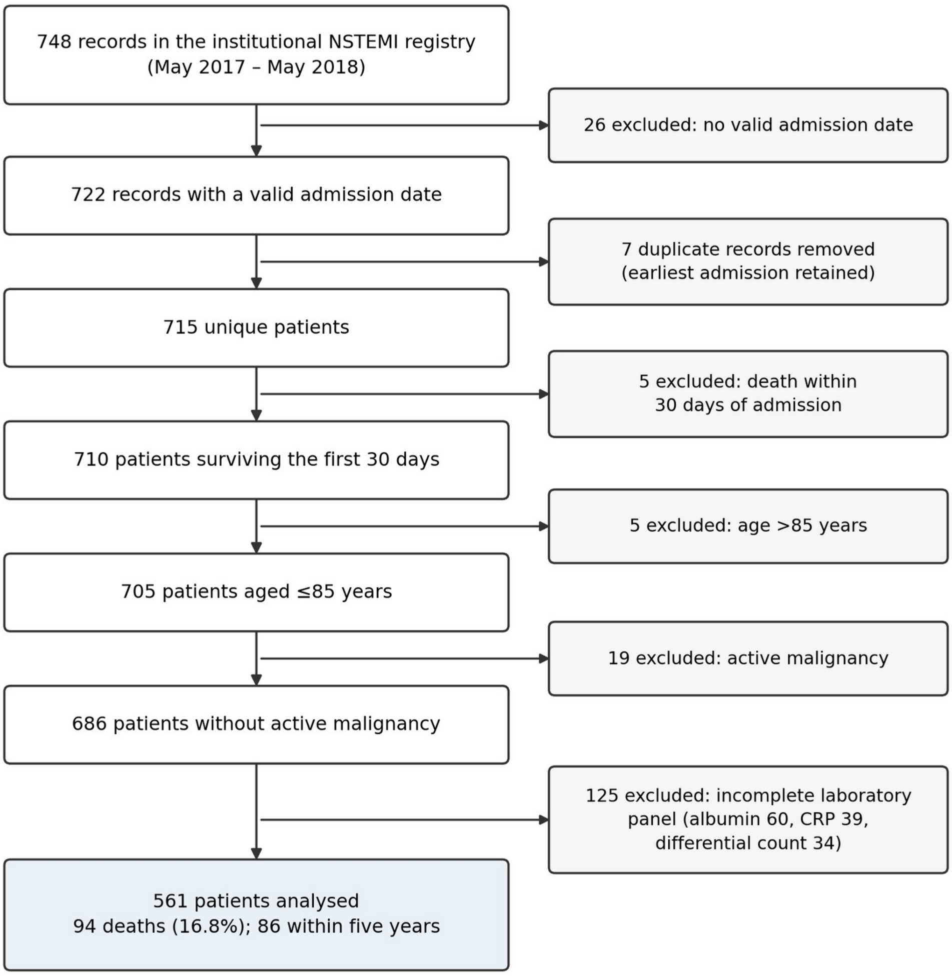

Supplementary Figure S1. Patient flow diagram

Supplementary Table S1. Comparison of analysed patients with patients excluded because of an incomplete laboratory panel

Variable	Analysed (n=561)	Excluded (n=125)	p
Age, years	59.4 ± 10.7	57.1 ± 12.0	0.050
Male sex	433 (77.2%)	95 (76.0%)	0.868
Hypertension	310 (55.3%)	64 (51.2%)	0.469
Diabetes mellitus	174 (31.0%)	37 (29.6%)	0.839
Prior myocardial infarction	133 (23.7%)	29 (23.2%)	0.996
LVEF, %	51.7 ± 9.7	52.6 ± 8.8	0.284
eGFR, mL/min/1.73 m^2	85.0 ± 20.6	86.7 ± 21.0	0.412
Revascularisation	375 (66.8%)	83 (66.4%)	1.000
All-cause mortality	94 (16.8%)	10 (8.0%)	0.020

Values are mean ± standard deviation or n (%). P values are from Welch independent-samples t tests for continuous variables and chi-square tests for categorical variables. Analyses use available values for each row; eGFR was available in 518 analysed and 122 excluded patients. No imputation was performed.

Supplementary Table S2. Marker-specific available-case association sensitivity analysis

Marker	n	Deaths	Adjusted HR	p	ΔC
HALP	553	87	1.28	0.037	-0.0015
Modified HALP	553	87	1.05	0.707	+0.0010
CALLY	518	84	1.34	0.006	+0.0070
CAR	551	84	1.42	0.001	+0.0110
CLR	569	89	1.24	0.036	+0.0042
PNI	553	87	1.31	0.025	-0.0012
NPAR	553	87	1.16	0.034	+0.0023
SII	606	92	1.26	0.030	+0.0025
SIRI	606	92	1.24	0.051	-0.0006
PIV	606	92	1.28	0.019	-0.0019
NLR	606	92	1.19	0.110	+0.0027
PLR	606	92	1.14	0.239	-0.0007
MLR	606	92	1.12	0.323	-0.0009

Each composite was analysed in all otherwise eligible patients with that marker and all clinical covariates observed. Hazard ratios are per 1-SD adverse change. ΔC denotes the apparent change in Harrell's C-index over the clinical model fitted in the same marker-specific sample. Marker-wise p values are nominal and were not adjusted for multiplicity.

Supplementary Table S3. Marker/reference-specific available-case CRP-plus-albumin decomposition

Marker	n	Deaths	Reference C	ΔC	Adjusted HR	p
HALP	522	84	0.7840	−0.0014	1.14	0.335
Modified HALP	522	84	0.7840	−0.0003	0.92	0.500
CALLY	518	84	0.7826	+0.0009	0.86	0.667
CAR	–	–	–	not estimable	–	–
CLR	522	84	0.7840	+0.0006	0.87	0.696
PNI	522	84	0.7840	+0.0001	1.00	0.978
NPAR	522	84	0.7840	+0.0007	1.17	0.414
SII	522	84	0.7840	−0.0005	1.17	0.196
SIRI	522	84	0.7840	−0.0002	1.04	0.784
PIV	522	84	0.7840	−0.0007	1.10	0.448
NLR	522	84	0.7840	−0.0002	1.09	0.482
PLR	522	84	0.7840	−0.0008	1.07	0.601
MLR	522	84	0.7840	+0.0011	0.92	0.526

For each composite, the analysis includes all otherwise eligible patients with that composite, CRP, albumin, outcome and all clinical covariates observed. The reference model contains the clinical covariates plus CRP and albumin entered separately. ΔC is the apparent change in Harrell's C-index after adding the composite. CAR is not estimable because log(CAR) is an exact linear combination of log(CRP) and log(albumin).

Supplementary Table S4. Sensitivity analyses

Analysis	n	Deaths	ΔC of CRP + albumin	Composite index p range
Primary (deduplicated, full follow-up)	518	84	+0.0106	0.20 – 0.99
Fixed five-year endpoint	518	76	+0.0156	0.27 – 0.97
Duplicate records retained	524	85	+0.0145	0.19 – 0.84
Non-obstructive coronary arteries excluded	431	73	+0.0102	0.16 – 0.99
Revascularisation omitted from the clinical model	518	84	+0.0133	0.18 – 0.87
Multivessel disease and revascularisation omitted from clinical model	518	84	+0.0152	0.19 – 0.86
Revascularisation strategy instead of any revascularisation (post hoc)[a]	518	84	+0.0108	0.19 – 0.98

ΔC of CRP + albumin denotes the change in the C-index when both are added to the clinical model. The p range refers to all twelve composite indices that are estimable over the

reference model in each analysis; CAR is not estimable in that model by construction. [a]Post hoc analysis in which any revascularisation was replaced by a three-level revascularisation strategy (no revascularisation; percutaneous coronary intervention of the culprit vessel only in multivessel disease; other revascularisation). The clinical C-index was 0.7728; every 2,000-resample bootstrap 95% confidence interval for the change in the C-index after adding each composite to this clinical model included zero.

Supplementary Table S5. Discrimination for all-cause mortality at one, three and five years

Marker	1 year (23 deaths)	3 years (48 deaths)	5 years (86 deaths)
CALLY	0.723 (0.610–0.836)	0.665 (0.578–0.753)	0.689 (0.623–0.756)
CLR	0.721 (0.611–0.831)	0.662 (0.575–0.749)	0.685 (0.618–0.751)
CAR	0.687 (0.564–0.810)	0.656 (0.569–0.743)	0.674 (0.607–0.740)
CRP	0.685 (0.569–0.801)	0.649 (0.563–0.735)	0.666 (0.600–0.733)
Albumin	0.658 (0.514–0.803)	0.645 (0.553–0.736)	0.635 (0.566–0.703)
HALP	0.655 (0.515–0.794)	0.610 (0.521–0.699)	0.644 (0.579–0.708)
NLR	0.646 (0.525–0.766)	0.631 (0.549–0.714)	0.651 (0.591–0.712)
NPAR	0.636 (0.499–0.772)	0.638 (0.551–0.725)	0.661 (0.599–0.723)
SII	0.621 (0.494–0.747)	0.613 (0.530–0.697)	0.640 (0.579–0.701)

Values are areas under the receiver operating characteristic curve with 95% confidence intervals. Mortality at each horizon was completely observed, so no censoring adjustment was required. Confidence intervals at one year are wide because only 23 deaths had occurred.

Supplementary Table S6. Continuous versus dichotomised CLR

Analysis	Estimate (95% CI)	p	ΔC over clinical model (95% CI)
Continuous, per 1 SD, unadjusted	HR 1.65	<0.001	–
Continuous, per 1 SD, adjusted	HR 1.32 (1.07–1.62)	0.010	+0.0063 (−0.0032 to +0.0255)
CLR ≥0.377, unadjusted	HR 3.22	<0.001	–
CLR ≥0.377, adjusted	HR 2.39 (1.49–3.83)	<0.001	+0.0133 (−0.0011 to +0.0361)
CLR ≥0.377, over clinical + CRP + albumin	HR 2.42	0.013	+0.0037

The threshold of 0.377 was identified by the Youden index in this cohort and is therefore exploratory and optimistic. Sensitivity was 68.6% and specificity 65.3% for five-year mortality. During full follow-up, mortality was 61 of 224 patients (27.2%) above the threshold and 33 of 337 (9.8%) below it. The reported ΔC confidence interval for the dichotomised CLR is conditional on this selected threshold and does not incorporate uncertainty from cut-point selection.
